# Supplementary material for: Do the Dietary Intakes of Pregnant Women Attending Public Hospital Antenatal Clinics Align with Australian Guide to Healthy Eating Recommendations?
Source: Nutrients. 2020 Aug 13;12(8):2438. doi: 10.3390/nu12082438 (PMC7468772; doi:10.3390/nu12082438)
Supplement: Supplementary file 1 [file nutrients-12-02438-s001.pdf]

Supplementary Table S1

THE UNIVERSITY OF
NEWCASTLE
AUSTRALIA

The association between diet quality during pregnancy and maternal and infant health and health care costs
Questionnaire

Thank you for completing this survey.

Please answer every question you can. If you are unsure about how to answer a question, mark the response for the closest answer to how you feel.

Please read the instructions above each question carefully. Some require you to only answer those options which are applicable to you. Other questions require you to mark one answer on each line.

You will be asked to provide your Medical record number, so please have it ready. Please remember that your details will remain confidential.

If you feel distressed now or throughout completing the survey and would like someone to talk to, you could ring Lifeline on 131 114 (local call) or NSW Mental Health access line 1800 011 511

Section A- About you

The following questions are about you:

**1. Date of birth**
Day / Month / Year
**2. What is the highest qualification you have completed (tick one)?**
☐ No formal qualifications
☐ School certificate (Year 10 or equivalent)
☐ Higher school certificate (Year 12 or equivalent)
☐ Trade/Apprenticeship (e.g. Hairdresser/Chef)
☐ Certificate/Diploma (e.g. childcare, technician)
☐ University Degree
☐ Higher University Degree (e.g. Grad Dip, Masters, PhD)

**3. Are you of Aboriginal or Torres Strait Islander origin?** *Circle one number only*
☐ No
☐ Aboriginal
☐ Torres Strait Islander
☐ Both Aboriginal and Torres Strait Islander
**4. In which country were you born?** *Circle one number only*
☐ Australia
☐ England
☐ New Zealand
☐ Other, please specify: ____
**5. What is your PRESENT marital status? (***Tick one)*
☐ Married
☐ Defacto
☐ Separated, but not divorced
☐ Divorced
☐ Widowed
☐ Never married
**6. Do you speak another language at home ? (***Tick one)ds*
☐ No I only speak English at home
☐ Yes<br>List other languages

**6. What is the average gross (before tax) income per week of yourself and your household (e.g. you and your partner, or you and your parents sharing a house)?** *Tick one for yourself and one for your household*	**You**	**House-hold**
No income	☐	☐
$1-$199 ($1-$10,399 annually)	☐	☐
$200-$299 ($10,400-$15,599 annually)	☐	☐
$300-$399 ($15,600-$20,799 annually)	☐	☐
$400-$599 ($20,800-$31,199 annually)	☐	☐
$600-$799 ($31,200-$41,599 annually)	☐	☐
$800-$999 ($41,600-$51,999 annually)	☐	☐
$1,000-$1,249 ($52,000-$64,999 annually)	☐	☐

$1,250-$1,499 ($65,000-$77,999 annually)	☐	☐
$1,500-$1,999 ($78,000-$103,999 annually)	☐	☐
$2,000 or more ($104,000 or more annually)	☐	☐
Don't know	☐	☐
Don't want to answer	☐	☐
I live alone (household income is the same as mine)		☐
**7. How do you manage on the income you have available? Tick one**		
☐ It is impossible		
☐ It is difficult all of the time		
☐ It is difficult some of the time		
☐ It is not too bad		
☐ It is easy		
**8. What is your postcode?**		

Section B- About your health

**1. Are you currently taking any medications or over-the-counter (including herbal tablets, multivitamins etc.)?** *(tick one)*
☐ Yes
☐ No *Go to Question 3*
**2. Please list the medications, herbal tablets or multivitamins:**
______________________________ ______________________________ ______________________________ __________
**3. Do you currently smoke tobacco products? (tick one)**
☐ Yes, daily
☐ Yes, at least once a week *Go to Section C*
☐ Yes, but less often than once a week *Go to Section C*
☐ No, not at all *Go to Section C*
**4. On an average day, how many cigarettes do you smoke?**
**5. How soon after waking up do you smoke?** *(tick one)*
☐ Within 5 minutes
☐ 6 to 30 minutes
☐ 31 to 60 minutes
☐ After 60 minutes
**6. Have you smoked at least 100 cigarettes or a similar amount of tobacco in your life?** *(tick one)*
☐ Yes
☐ No
☐ Not sure
**7. Have you been diagnosed or treated for any of the following health conditions:**
☐ High blood cholesterol or triglyceride levels
☐ High blood pressure (hypertension)
☐ Preeclampsia
☐ Polycystic Ovarian Syndrome (PCOS)

☐ Obesity
☐ Type 2 diabetes (NIDDM or adult onset diabetes)
☐ Pre-diabetes / Impaired Glucose Tolerance or Impaired Fasting Glucose
☐ Gestational Diabetes
☐ Heart conditions
☐ None of these conditions
**8. How many children do you have (please include all children under the age of 18 years living in the household)**
**9. Please enter the date of each child's birth in the following (DD/MM/YY)**
Child 1
Child 2
Child 3
Child 4
Child 5
Child 6
Child 7

Section C- About your pregnancies

**1. How many weeks pregnant are you?**

**2. What was your weight before your current pregnancy?**
**3. When was this weight measure collected?**
**4. How much weight have you gained during your current pregnancy?**
**5. How much weight did you gain during each of your pregnancies (kilograms)**
Child 1
Child 2
Child 3
Child 4
Child 5
Child 6
Child 7
**6. What is your height?**

Section D: Pregnancy healthcare

**1. Have you receive any advice from health professionals on diet during pregnancy?**
☐ Yes
☐ No
☐ Unsure
**2. Did you receive any information on weight loss after the birth of your baby from your most recent pregnancy?**
☐ Yes
☐ No

☐ Not applicable as this is my first pregnancy
**3. Have you had a dental check up in the last 6 months?**
☐ Yes
☐ No (go to question 4)
**4. Have you had a dental check up in the last 12 months?**
☐ Yes
☐ No
**5. In the past 12 months was there any time when you ran out of food and couldn't afford to buy more?**
☐ Yes → Go to Q6
☐ No
**6. When this happened, did you go without food?**
☐ Yes
☐ No

Section D- other diet questions

These next questions are on foods that are not included in the Australian eating survey.

Think about what you ate in the last 3 to 6 months when you answers these questions

How often do you have the following foods?

	Never	Less than	1-3 per month	Once per week	2-4 per week	5 or more per week

		once per month				
Pate or meat spreads						
Salads (pre prepared) or (pre packaged) from salad bars or smorgasbord						
Soft serve ice cream						

In order for us to obtain your medical record and ObsetriX Data can you please complete the following:

First Name:

Middle Name:

Surname

Medical record number:

Doctors Name :

Expected due date:

What hospital are you booked into to have your baby? (choose one)

- ☐ Belmont Hospital
- ☐ Glen Innes Hospital
- ☐ Gloucester Soldier's Memorial Hospital
- ☐ Gunnedah Hospital
- ☐ Inverell Hospital
- ☐ John Hunter Hospital
- ☐ Maitland Hospital
- ☐ Manning Hospital
- ☐ Moree Hospital
- ☐ Muswellbrook Hospital
- ☐ Narrabri Hospital
- ☐ Scott Memorial Hospital - Scone

☐ Singleton Hospital
☐ Tamworth Hospital

Thank you for completing this survey, please click on this link for the next section: Australian Eating Survey

Supplementary Figure S1: Recruitment for pregnant women attending the John Hunter Hospital Antenatal Clinic, in Newcastle, NSW.

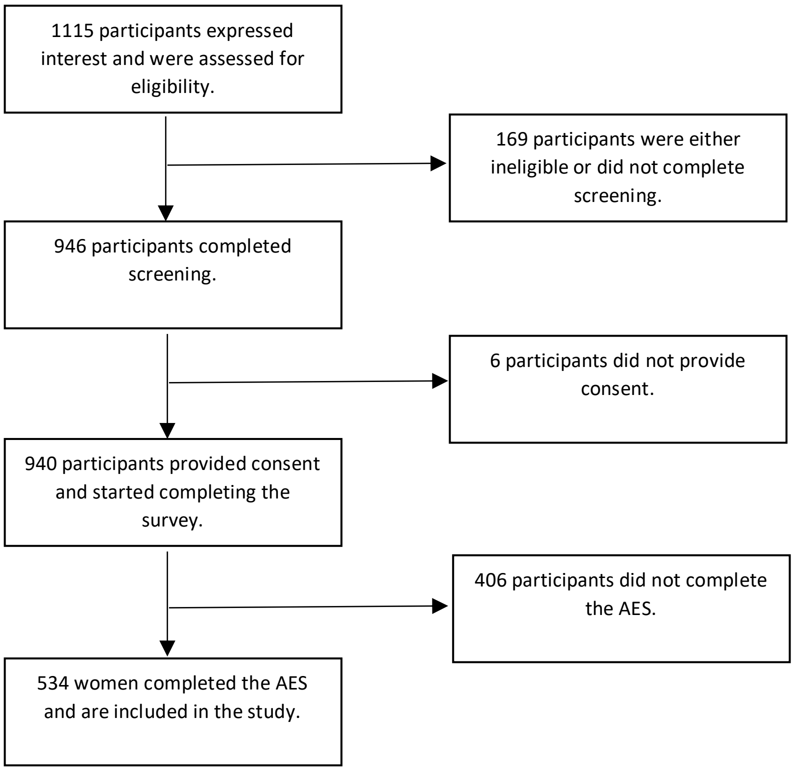

Flow diagram for the process of recruitment for pregnant women attending the John Hunter Hospital antenatal clinic.

Supplementary Table S2: Daily food consumption differences in pregnant women from the John Hunter Hospital antenatal clinic, by BMI category.

		**BMI <18.5 kg/m² (n=20)** Median (IQR)	**BMI 18.5-24.9 kg/m² (n=171)** Median (IQR)	**BMI 25-29.9 kg/m² (n=80)** Median (IQR)	**BMI ≥30 kg/m² (n=175)** Median (IQR)	**Missing BMI (n=88)** Median (IQR)
**Food group servings (servings/day)**	**AGHE (serves/day)**					
Breads and cereals	8.5	2.8 (1.9-3.3)	3.0 (2.1-4.1)	2.6 (1.7-3.6)	2.4 (1.5-3.4)	2.5 (1.3-3.8)
Fruit	2	1.7 (1.4-2.6)	2.0 (1.2-2.9)	1.8 (1.7-2.4)	1.4 (0.8-2.1)	1.7 (0.8-2.6)
Vegetables/legumes	5	3.6 (2.9-4.5)	3.9 (3.0-5.4)	4.0 (2.0-5.0)	3.8 (2.9-5.2)	3.7 (2.5-5.0)
Dairy/alternatives	2.5	1.1 (0.7-1.8)	1.4 (0.9-1.9)	1.4 (0.9-1.8)	1.2 (0.6-1.8)	1.1 (0.6-1.8)
Meat/alternatives	3.5	2.0 (1.3-2.8)	2.4 (1.7-3.2)	2.3 (1.6-3.3)	2.3 (1.7-3.3)	2.2 (1.6-3.2)
**Macronutrients**	**NRVs (unit/day)**					
Energy (kJ) With dietary fibre	-	8125 (6948-8985)	8680 (6802-10123)	8240 (6468-9966)	7658 (6208-9360)	7681 (5516-10510)
CHO (% E)	AMDR 45-65%	46 (43-50)	46 (42-49)	46 (39-50)	44 (40-50)	45 (41-52)
Protein (% E)	AMDR 15-25%	16 (15-18)	18 (16-19)	17 (16-20)	18 (17-21)	18 (16-20)
Fat (% E)	AMDR 20-35%	37 (34-39)	37 (34-39)	37 (34-41)	38 (35-41)	37 (33-40)
Sat. Fat (% E)	<10%	14 (12-15)	14 (12-15)	15 (13-16)	14 (13-16)	14 (12-16)
Omega 3 (mg)	-	131.5 (96.5-223.5)	176.3 (121-275)	179.8 (116.0-246.5)	169.5 (108-252)	169.4 (84.9-259.2)
Fibre (g)	28	27.5 (24.2-29.5)	28.2 (22-36)	25.9 (21-32)	23.4 (18-30)	24.7 (17.1-35.4)
% energy from core foods	-	66 (50-73)	69 (62-78)	64 (57-73)	67 (58-75)	65 (56-75)
% energy from non-core foods	-	34 (28-51)	31 (23-38)	36 (27-75)	33 (26-42)	36 (25-45)
**Micronutrients**	**NRVs (unit/day)**					
Thiamin (mg)	EAR 1.2	1.2 (1.0-1.8)	1.6 (1.0-1.8)	1.5 (1.1-2.0)	1.4 (1.1-1.8)	1.4 (1.1-2.0)
Riboflavin (mg)	EAR 1.2	1.8 (1.3-2.4)	2.1 (1.6-2.6)	2.0 (1.5-2.5)	1.8 (1.4-2.4)	1.8 (1.3-2.5)
Niacin equivalents (mg)	EAR 14	32.1 (29.1-39.2)	38.4 (29.6-44.7)	35.7 (29.7-44.6)	34.8 (26.9-46.0)	35.3 (27.0-46.3)
Vitamin C (mg)	EAR 40	177.1 (111.1-212.7)	172.3 (122.9-217.8)	170.0 (113.5-228.9)	153.8 (105.5-215.7)	154.0 (103.5-252.6)
Dietary folate equivalents (µg)	EAR 520	473.6 (390.5-639.2)	542.8 (457.4-669.8)	548.7 (424.3-682.6)	504.0 (380.2-643.5)	591.1 (382.6-672.6)
Retinol equivalents (µg)	EAR 550	760.4 (606.7-1123.0)	998.0 (732.7-1281.5)	893.4 (691.1-1161.7)	859.9 (562.5-1116.5)	891.7 (597.4-1176.1)
Magnesium (mg) 19-30 years old	EAR 290	354.9 (282.3-375.8)	379.5 (327.5-463.8)	359.6 (309.7-443.6)	336.0 (276.2-423.4)	328.6 (253.7-450.5)
Phosphorus (mg)	EAR 580	1283.1 (1152.2-1504.8)	1451.1 (1108.2-1712.5)	1346.0 (1107.8-1676.7)	1292.0 (997.8-1613.6)	1290.4 (920.9-167.1)
Calcium (mg)	EAR 840	779.7 (567.7-959.3)	831.6 (638.7-1008.2)	803.0 (618.2-975.4)	731.7 (501.2-926.0)	688.6 (446.0-954.7)
Iron (mg)	EAR 22	9.2 (7.9-10.6)	11.2 (9.0-13.3)	9.9 (8.0-12.0)	9.3 (7.4-12.0)	9.5 (7.4-13.2)
Zinc (mg)	EAR 9.0	9.8 (9.1-11.9)	11.4 (9.0-13.6)	10.8 (8.9-13.2)	10.8 (8.2-13.5)	10.1 (7.5-14.0)
Sodium (mg)	AI 460-920	1762.4 (1402.2-2102.4)	1727.2 (1355.1-2161.2)	1772.5 (1415.3-2971.9)	1768.8 (1301.0-2218.6)	1666.2 (1243.3-2292.6)
Iodine (ug)	EAR 160	133.5 (98.7-149.8)	128.8 (99.1-158.1)	127.1 (98.0-154.8)	116.9 (83.4-153.3)	111.8 (77.1-159.6)
Potassium (mg)	AI 2800	3177.0 (2768.5-3516.6)	3472.9 (2531.8-3892.2)	3311.8 (2639.2-3854.6)	2928.9 (2372.7-3753.0)	3026.6 2334.5-4020.0

Supplementary Table S3: Percentage of participants meeting AGHE and NRV recommendations, by BMI category.

		**BMI <18.5 kg/m² (n=20)** n(%)	**BMI 18.5-24.9 kg/m² (n=171)** n(%)	**BMI 25-29.9 kg/m² (n=80)** n (%)	**BMI ≥30 kg/m² (n=175)** n (%)	**Missing BMI (n=88)** n (%)
**Food group servings (servings/day)**	**AGHE (serves/day)**					
Breads and cereals	8.5	0 (0)	0 (0)	0 (0)	0 (0)	3 (3)
Fruit	2	9.0 (45)	86 (50)	26 (33)	49 (28)	34 (39)
Vegetables/legumes	5	3.0 (15)	53 (31)	21 (26)	43 (25)	22 (25)
Dairy/alternatives	2.5	4.0 (20)	23 (14)	7 (9)	29 (17)	10 (11)
Meat/alternatives	3.5	3.0 (15)	26 (15)	13 (16)	39 (22)	16 (18)
**Micronutrients**	**NRVs (unit/day)**					
CHO (% E)	AMDR 45-65%	13 (65)	103 (60)	47 (59)	79 (45)	45 (51)
Protein (% E)	AMDR 15-25%	15 (75)	146 (85)	66 (83)	158 (90)	70 (80)
Fat (% E)	AMDR 20-35%	7 (35)	62 (36)	27 (34)	51 (29)	30 (34)
Sat. Fat (% E)	<10%	2 (10)	17 (10)	4 (5)	6 (3)	10 (11)
Fibre (g)	28	7 (35)	87 (51)	32 (40)	51 (29)	34 (39)
**Micronutrients**	**NRVs (unit/day)**					
Thiamin (mg)	EAR 1.2	10 (50)	130 (76)	55 (69)	113 (65)	55 (63)
Riboflavin (mg)	EAR 1.2	16 (80)	156 (91)	68 (85)	145 (83)	70 (80)
Niacin equivalents (mg)	EAR 14	20 (100)	170 (99)	77 (96)	173 (99)	83 (94)
Vitamin C (mg)	EAR 40	20 (100)	171 (100)	78 (98)	170 (97)	87 (99)
Dietary folate equivalents (µg)	EAR 520	7 (35)	94 (55)	45 (56)	83 (47)	43 (49)
Retinol equivalents (µg)	EAR 550	16 (80)	152 (89)	67 (84)	136 (78)	69 (78)
Magnesium (mg) 19-30 years old	EAR 290	14 (70)	141 (83)	64 (80)	116 (66)	52 (59)
Phosphorus (mg)	EAR 580	20 (100)	169 (99)	77 (96)	169 (97)	81 (92)
Calcium (mg)	EAR 840	7 (35)	84 (49)	35 (44)	61 (35)	28 (32)
Iron (mg)	EAR 22	1 (5)	4 (2)	0 (0)	0 (0)	2 (2)
Zinc (mg)	EAR 9.0	15 (75)	128 (75)	60 (75)	116 (66)	59 (67)
Sodium (mg)	AI 460-920	1 (5)	7 (4)	6 (8)	10 (6)	10 (11)
Iodine (ug)	EAR 160	3 (15)	39 (23)	48 (60)	39 (22)	22 (25)
Potassium (mg)	AI 2800	15 (75)	128 (76)	56 (70)	99 (57)	51 (58)
